# Red Cross Work in Turkey

**Published:** 1913-05-17

**Authors:** 


### RED CROSS WORK IN TURKEY.

### The Experience of Two "Lay Helpers."

With the return of the many units which the British Red Cross Society equipped and despatched to the several combatant nations in the Balkan War, we may expect at no distant date to see in print many accounts of their strange experiences in camp and quarters. Most of these, we may expect, will deal with the war from a medical, or at least, from a semi-medical standpoint; but one of the earliest of them,* which has already appeared, is written with a conspicuous lack of any such bias, and narrates the experiences of two Red Cross—or rather Red Crescent—men who had no technical training and remained throughout "lay helpers" in the work of their unit.

Quite on the spur of the moment, seemingly, Mr. Duncan-Johnstone and a brother Scot made up their minds to volunteer for Red Cross duty in the East; and were both selected by Sir Frederick Treves for service with the Turkish contingent. A good many of their comrades were medical students, and no doubt this accounted for the selection of the two authors (for Mr. Lyon contributes the sketches which illustrate his companion's narrative) as general duty orderlies. They seem, however, to have had at times a fair share of nursing work to do when the cholera patients and hundreds of wounded Turks were lying in the streets of Constantinople practically uncared for. Evidently the two Scots were the right kind of handy men for their job, for later on Mr. Duncan-Johnstone found himself camp-sergeant-major of a cholera hospital. But before this latter, the closing stage of the work of their unit, they had seen many strange sights, first of all in Constantinople itself and later in a dressing station a little way behind the celebrated Tchataldja lines. In all of these places the author's performances upon the bagpipes seem to have created great enthusiasm and admiration; and it is quite easy to believe that his proficiency upon the instrument materially soothed the savage Kurds, Turks, Albanians, and other soldiers of the crescent, and smoothed many rough passages for the whole British contingent.

In Constantinople, where the units arrived soon after the Lule Burgas disasters, work in plenty lay to hand. With equipment very largely improvised out of anything that came handy, the units did their level best to cope with the wounded from Kirk Kilisse, beginning to arrive from the front where they had been wounded three weeks before. These unfortunate wretches had in most cases received no attention since the battle, and their wounds were in a state too horrible for words to depict. A large proportion of them died on the way, and of those who did get to Constantinople a great number were in a dying condition. Add to this the ravages of cholera, just commencing to spread like fire in tow among the troops, and it will be realised that the Red Crescent men's initiation into the horrors of war was a vivid and gruesome affair.

The Turkish medical service was to all intents non-existent at the outbreak of hostilities; and what there was of it was non-efficient and disorganised. Probably the same was true to a large degree of the rest of the army; but it might have been supposed that at Constantinople of all places, the lessons of medical and hospital efficiency would have been learnt. For Scutari is only a gunshot away, across the Bosphorus, where Florence Nightingale's work was done during the Crimean campaign. Surely this ought to have been an object lesson; but it was not learnt, and but for the various auxiliary agencies sent to help them by the humanity of other nations and alien creeds, the plight of the Turkish army would indeed have been desperate.

### A Typical Case.

One example must suffice to show the sort of case with which the units had to deal. A man was brought to them on a stretcher, from which he had not been lifted since his wounds a fortnight before. He had a shrapnel wound in the calf of the leg, and another in the back; and was suffering from acute dysentery. The condition of the poor man can be better imagined than described; indeed, we doubt whether even imagination can picture it without some previous experience of like horrors to draw upon.

On the whole it is evident that the two volunteers enjoyed their experiences, though they had some terribly rough times. By reading between the lines, it is also easy to make out that some of their fellow-orderlies and dressers were misfits for the job. Of the medical officers under whose command they served not very much is said; but three or four are mentioned in terms of eulogy, which may or may not mean that some of the others were less esteemed. Of technical medical and surgical details the author very wisely steers clear. There is a disposition to attribute to the Turkish doctors a strong jealousy of the foreign Red Cross missions: this charge was also made by those who served in Tripoli. Another outstanding impression left upon the author by his experiences is the incapacity of the Turks to organise any form of co-ordinated effort in any branch of work. His experiences amongst them certainly bear out such a conclusion; they are told without any pretence at literary art, but in a pleasant, straightforward narrative which was well worth publishing, and will, we do not doubt, attract many readers who would not care about the more circumstantial and medical descriptions of the surgeons and physicians sent out by the Red Cross Society.

* *With the British Red Cross in Turkey.* By A. Duncan-Johnstone. James Nisbet and Co. Price 5s. net.

